# Remote Analysis and Transmission System of Electrocardiogram in Prehospital Setting; a Diagnostic Accuracy Study

**DOI:** 10.22037/aaem.v10i1.1399

**Published:** 2022-01-01

**Authors:** Elmira Almukhambetova, Murat Almukhambetov, Abdugani Musayev, Ainur Yeshmanova, Vildan Indershiyev, Zhadira Kalhodzhaeva

**Affiliations:** 1 Department of Emergency and First Aid, Asfendiyarov Kazakh National Medical University, Almaty, Kazakhstan.

**Keywords:** Cardiovascular system, cardiovascular diseases, diagnosis, quality of health care, health services administration

## Abstract

**Introduction:**

One of the trends in the development of medical technologies is considered to be telemedicine. This study aimed to evaluate the accuracy of a remote electrocardiogram (ECG) analysis and transmission system in prehospital setting.

**Methods:**

In this cross-sectional study, the data of 19,265 ECGs was gathered from emergency medical service (EMS) database of Almaty city, Kazakhstan, from 2015 to 2019. All ECGs were recorded in the prehospital setting by a paramedic, using "Poly-Spectrum" ECG recording device. Subsequently, all ECGs were sent to the cardiologist for interpretation and the findings were compared between software and cardiologist.

**Results:**

19,265 ECGs were registered. The average time from taking ECGs to receiving an expert’s conclusion was 9.2 ± 2.5 minutes. The medical teams were called in 17.9% of cases after paramedic ECG recording; however, in the rest of the cases there was no need to call those teams. Using the device reduced the number of visits of specialist teams.

The overall sensitivity, specificity, and accuracy of ECG analysis device in diagnosis of ECG abnormalities were 83.8% (95%CI: 82.6 – 84.9), 95.5% (95%CI: 95.1 – 95.8), and 93.3% (95%CI: 92.9 – 93.7), respectively.

**Conclusion:**

The findings of this study showed the 93.3% accuracy of automatic ECG analysis device in interpretation of ECG abnormalities in prehospital setting compared with the cardiologist interpretations. Using the device causes a decrease in the number of cardiologist visits needed as well as reduction in cost and elapsed time.

## 1. Introduction

One of the trends in development of medical technologies is considered to be telemedicine, the main goal of which is to create conditions to make the consultation of highly qualified experts easily accessible to ordinary citizens (1, 2). 

Considering the high prevalence and burden of cardiovascular diseases, the importance of a simple and accessible electrocardiography (ECG) analysis tools in prehospital settings is clear (3, 4). Thanks to the development of computer technologies, communication networks and the internet have made it possible to register an ECG anywhere and share it over long distances (5, 6).

The first experiments of ECG transmission over a significant distance took place at the beginning of the 20th century (7). In 1905, W. Einthoven transmitted an ECG at a distance of about 1.5 kilometers (8). The method of remote analysis and transmission of ECG began to spread in the 1960s with the emergence of technical capabilities that made it possible to achieve the sufficient quality of ECG reception (9-12).

In some cases, the description and interpretation of ECGs cause difficulties for paramedics (13, 14). Calling a specialized medical team to assist the paramedics in deciphering "difficult-to-analyze" ECG is economically and temporally unjustifiable. Using the remote analysis and transmission systems of ECG at prehospital settings could be helpful in this regard. This study aimed to evaluate the accuracy of a remote analysis and transmission system of ECG in the prehospital setting. 

## 2. Methods

***2.1 Study design and setting***

In this diagnostic accuracy study, the data of 19,265 ECGs of adult patients (aged ≥18 years) was gathered from EMS database of Almaty city, Kazakhstan, from 2015 to 2019. All ECGs were recorded in the prehospital setting by a paramedic and using the "Poly-Spectrum" ECG recording device, and then remotely transmitted to the cardiologists.

The findings of the automatically obtained analysis from the system were compared with the conclusions made by 19 experienced physicians working in the cardiology center (from 6 to > 30 years). The doctor previewed the ECGs and further excluded unnecessary artifacts performed by automatic analysis to achieve high accuracy of the final results. Consequently, system and specialist reports were compared with each other.

***2.2 About the system***

The system for remote analysis and transmission of ECG included the following parts:

- 12- lead ECG registration, as well as transmitting devices that allow paramedic teams to share ECGs immediately after recording and monitor the patient's condition during transportation to a medical institution. 

- receiving and transmitting devices suitable for recording ECGs placed in the cardiology remote consultation point, on the emergency medical service (EMS) station and the admission department of the city cardiology center. These devices help specialists to consult people and reach a syndromic conclusion in a couple of seconds online and by phone. 

Moreover, specialists analyzed the ECGs in complicated clinical cases such as emergency hospitalization and thrombolytic therapy. They also provided advisory support and accurate recommendations to health professionals who transmitted the ECGs in order to monitor the patient at the pre-hospital setting. Indications for using the mentioned system were as follows:

- The presence of clinical manifestations of acute coronary syndrome (unstable angina, heart attack) 

- Acutely formed life-threatening condition or hemodynamic disruption

- Tachy/brady dysrhythmia in case of being unable to analyze ECGs on the scene 

***2.3 Data gathering***

In this diagnostic accuracy study, the data of 19,265 ECGs of adult patients (aged ≥18 years) was gathered from EMS database of Almaty city, Kazakhstan. All ECGs were recorded in prehospital setting by a paramedic and using the "Poly-Spectrum" ECG recording device and then remotely transmitted to the cardiologists. Consequently, they analyzed the ECGs to make the conclusion more exact.

The results of the ECG analysis and recommendations were stored and registered in the surveillance log. 

***2.4 Statistical analysis***

Sensitivity, specificity, and accuracy were evaluated in order to establish the diagnostic capabilities of the tests. Assessment and presentation of these indicators were carried out by calculating the 95% confidence interval using the statistical analysis package SPSS 13.0 for Windows.

***2.5 Ethical considerations***

The research corresponded to Declaration of Helsinki, developed by the World Medical Association. Permission or approval of the ethics committee was not required, because the publication describes a retrospective study, only a statistical analysis of available patient data was carried out. 

## 3. Results

**Figure 1:**
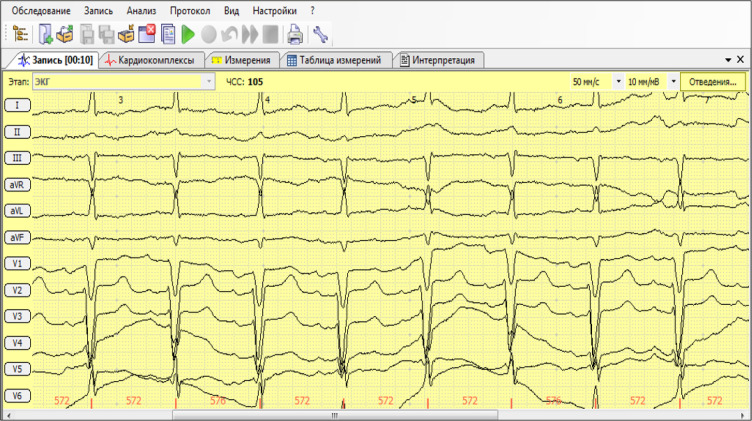
Example of a 73 years old patient's ECG recorded by a paramedic in 12 leads and sent to a specialist. Interpretation of ECG by "Poly-Spectrum" recording device, which was confirmed by a specialist:

19,265 ECGs were registered and stored in the database during the overall period of application. The average time from taking ECGs to receiving an expert’s conclusion was 9.2 ± 2.5 minutes.  Figure 1 shows a sample of transmitted ECG, diagnosis, and recommendation of specialist. The medical teams were called in 17.9% of cases after paramedic ECG recording; however, in the rest of the cases calling those teams was not required. The introduction of devices for recording and transmitting ECGs had its economic effect by reducing the number of visits of specialist teams. Owing to that, 52,542,432 tenge (140000 $) was saved from being lost in vain only by 2016.

***3.1 Accuracy of ECG interpretation device***

According to the specialists' interpretations, ECGs were analyzed to be normal in 16,992 (88.2%) patients (53% male) and had at least one abnormality in 7,086 (36.8%) cases. Table one shows the frequency of ECG abnormalities and screening performance characteristics of the device in diagnosis of each abnormality. The overall sensitivity, specificity, and accuracy of ECG analysis device in diagnosis of ECG abnormalities were 83.8% (95%CI: 82.6 – 84.9), 95.5% (95%CI: 95.1 – 95.8), and 93.3% (95%CI: 92.9 – 93.7), respectively.

## 4. Discussion

Based on the findings of the present study, the sensitivity, specificity, and overall accuracy of the automatic ECG analysis in the prehospital setting were 83.8%, 95.5%, and 93.3%, respectively. 

Taking into consideration a review of previous studies on various automatic ECG analysis programs by Lyon, Aurore et al., the sensitivity ranged from 75.9% to almost 100%, depending on the specific conclusion and method of electrocardiogram analysis (15).

Also, in the study of de Chazal P. et al., the sensitivity was 75.9% for determining supraventricular extrasystoles, and 77.7% for ventricular extrasystoles (16).

The diagnostic accuracy indicators for the blockage of the left leg of the His bundle, right bundle, extrasystole, atrial fibrillation, ventricular fibrillation, sinus node weakness syndrome, and normal ECG portrayed in a study by Niwas et al., were near 99% (17).

Remote analysis and advisory support in making diagnostic and clinical decisions based on the interpretation of electrocardiograms help in using the practical clinical experience of highly qualified consultants where it is needed the most.

During transportation to a medical facility, the program also allows monitoring of the patient’s condition and ensures that the medical facility is ready to receive a patient with an urgent condition, inasmuch as emergency revascularization in acute myocardial infarction.

To record and decipher the ECG in an ordinary situation, the patient will have to get to an outpatient clinic or hospital, where the ECG will be further registered and analyzed. The great deal of effort, money, and time are required from the patient. As a result, the implementation of emergency care may be delayed. Additionally, material costs for fuel and support of sanitary transport are also required to achieve the goal. 

With the introduction of a system for remote analysis of ECGs, all these problems are automatically solved, and it becomes possible to receive highly qualified diagnostic assistance in the conditions of the pre-hospital stage. Besides, the direct economic effect of the mass introduction of ECGs recording and transmission devices is obvious, as the number of visits of specialized intensive care teams decrease.

Generally, during usage of remote ECG analysis, various problems occurred in about 0.3% of cases due to obtaining an "atypical" electrocardiogram (artifacts, etc.), which can be the result of incorrect positioning of the electrodes, patient's muscle tremors, hardware errors, the performer’s inexperience, and software failures. As a consequence, re-registration and transmission of ECGs are often required in these cases. 

This method is recommended to be implemented in practical healthcare for early diagnosis and assistance, which can lead to an improvement in the health indicators of the population. 

Sinus regular rhythm with a heart rate of 105 beats per minute; Tachycardia; Deviation of the electrical axis of the heart to the left; Probably a lower myocardial infarction, the stage of scarring; Possible anterior myocardial infarction, subacute stage; and Left ventricular hypertrophy (LVH).

The specialist recommended comparing it with previous ECGs and contrasting them with the symptoms and calling the intensive care team. 

**Table 1:** Sensitivity and specificity of the electrocardiogram (ECG) analysis device in diagnosis of different ECG abnormalities compared with cardiologist’s interpretation

Abnormality	N (%)	TP	FP	FN	TN	Sensitivity	Specificity	Accuracy
Atrial fibrillation	1387 (7.2)	1311	607	76	17878	94,5 (93,2; 95,6)	96,7 (96,5; 97,0)	96,6 (96,3; 96,8)
Atrial flutter	139 (0.7)	108	56	31	19126	77,7 (70,1; 83,8)	99,7 (99,6; 99,8)	99,6 (99,5; 99,6)
LVH	4527 (23.5)	3678	1353	849	14738	81,3 (80,1; 82,4)	91,6 (91,2; 92,0)	89,3 (88,9; 89,7)
AV block	559 (2.9)	473	195	86	18706	84,6 (81,4; 87,4)	98,9 (98,8; 99,1)	89,3 (88,9; 89,7)
Extra-systole	1888 (9.8)	1659	935	229	17377	87,9 (86,3; 89,3)	94,9 (94,6; 95,2)	94,2 (93,9; 94,6)
RBBB	1522 (7.9)	1397	848	125	17743	91,8 (90,3; 93,1)	95,4 (95,13; 95,73)	95,2 (94,9; 95,5)
LBBB	2639 (13.7)	2411	1258	228	16626	91,4 (90,2; 92,4)	92,9 (92,6; 93,3)	92,8 (92,4; 93,1)
Ischemia	1291 (6.7)	1168	489	123	17974	90,5 (88,8; 92,0)	97,4 (97,1; 97,6)	96,9 (96,7; 97,1)
Lower MI	167 (6.8)	148	84	19	19098	88,6 (82,9; 92,6)	99,6 (99,5; 99,6)	99,5 (99,4; 99,6)
Anterior-lateral MI	153 (0.9)	138	78	15	19112	90,2 (84,5; 94,0)	99,6 (99,5; 99,7)	99,5 (99,4; 99,6)
Sinus rhythm	16992 (88.2)	17817	283	782	1448	95,8 (95,5; 96,1)	83,7 (81,8; 85,3)	94,8 (94,5; 95,1)
Normal	12179 (63.2)	7439	2920	4740	11826	61,1 (60,2; 61,9)	80,2 (79,6; 80,8)	71,6 (71,0; 72,1)

All measures are presented with 95% confidence interval. N: number; FP: false positive; FN: false negative; TP: true positive; TN: true negative; LVH: left ventricular hypertrophy; AV: atrioventricular; RBBB: right bundle brunch block; LBBB: left bundle brunch block; MI: myocardial infarction.

## 5. Limitations

As with any cross-sectional study, there was a risk of selection bias. The research was held at the particular region – Almaty city (Republic of Kazakhstan). 

## 6. Conclusion

The findings of this study showed the 93.3% accuracy of automatic ECG analysis device in interpretation of ECG abnormalities in prehospital setting compared with the cardiologist interpretations. Using the device causes a decrease in the number of specialized intensive care teams’ visits.

## 7. Declarations:

### 7.1 Acknowledgments

The authors of the article express their sincere gratitude to the management and staff of the Almaty Ambulance Service for their assistance in carrying out this work.

### 7.2 Authors' contributions

The contribution of each author is in the analytical search for scientific publications, writing the article and approving the content.

### 7.3 Conflict of interest

 No potential and actual conflicts of interest were present during our investigation.

### 7.4 Availability of data

The data of medical records of patients used in the publication are available only to healthcare workers of the Republic of Kazakhstan, who are working on the electronic resource of the complex medical information system called “Damumed” (https://alm.dmed.kz/Authentication/Authentication/SignIn ?ReturnUrl=%2F) 

### 7.5 Funding and supports

 None
